# Supplementary figures and images for: A hidden battle in the dirt: Soil amoebae interactions with Paracoccidioides spp
Source: PLoS Negl Trop Dis. 2019 Oct 7;13(10):e0007742. doi: 10.1371/journal.pntd.0007742 (PMC6797224; doi:10.1371/journal.pntd.0007742)

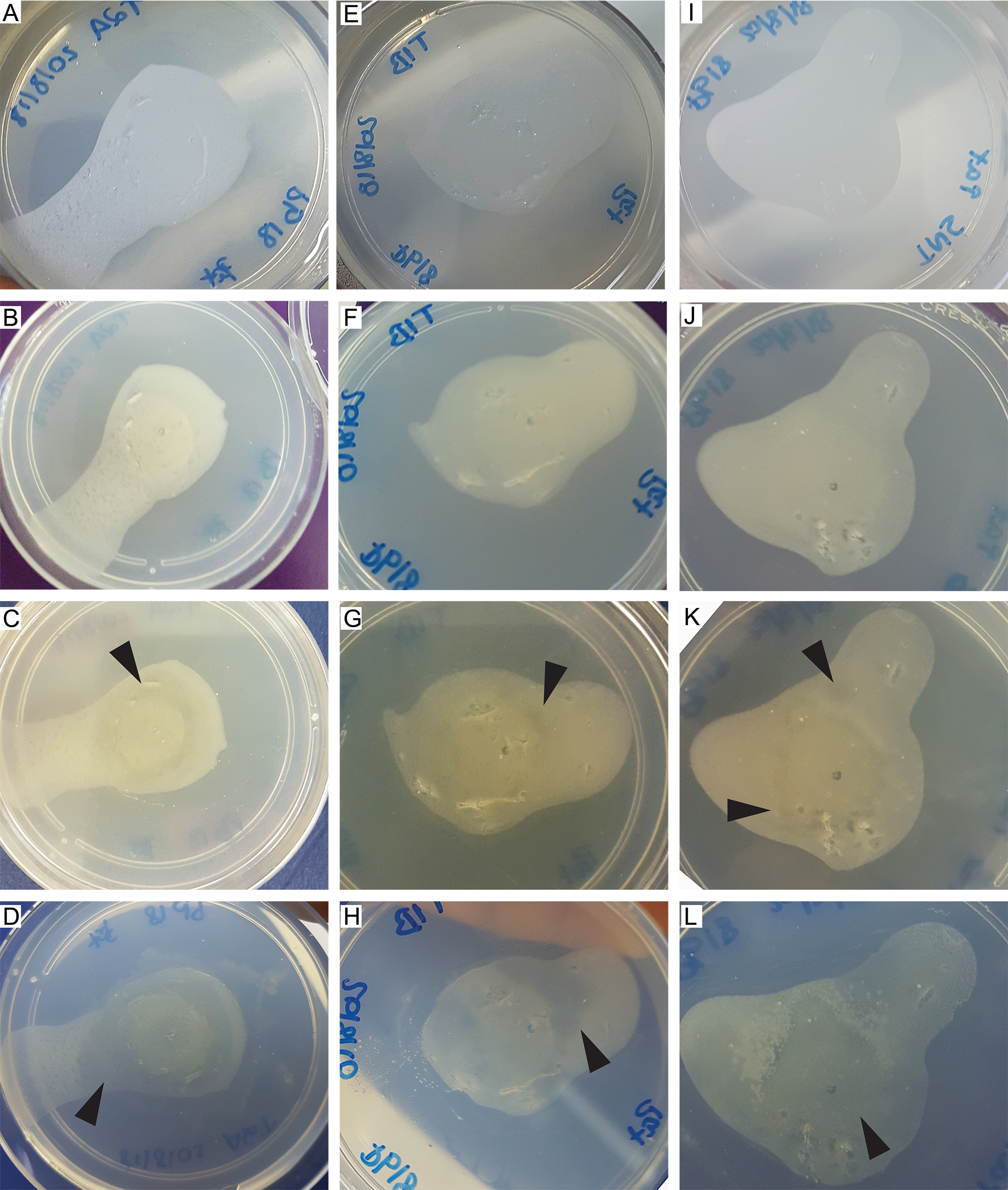

Supplement: S1 Fig — A suspension of 1.5 x10^7^ P. brasiliensis Pb18 cells was plated onto non-nutrient agar and spotted with 104 cells of Acanthamoeba spp (panels A-D), A. spelaea (panels E-H) or V. vermiformis (panels I-L) in 10-microlitre aliquots directly in the middle of the fungal cell lawn. The plates were incubated at 25°C for 19 days and inspected for the formation of lysis plates and fungal cell digestion at day 1 (panels A, E, I), day 3 (panels B, F, J), day 7 (panels C, G, K) and day 19 of interaction (panels D, H, L). Black arrowheads depict regions of fungal clearance. (TIF) [file pntd.0007742.s001.tif]

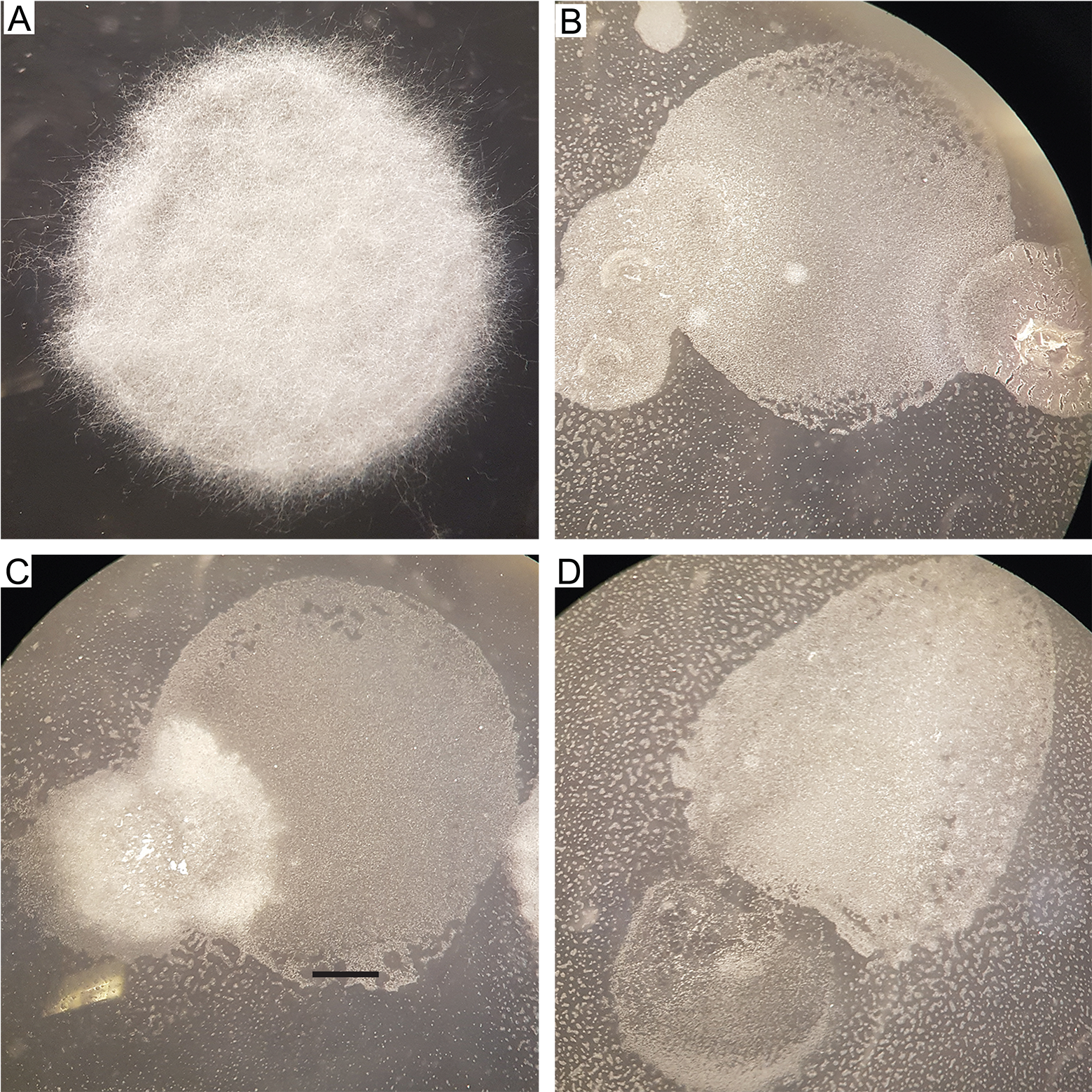

Supplement: S2 Fig — Five microliters of a suspension of 1.5 x10^7^ P. brasiliensis Pb18 cells were plated onto non-nutrient agar, spotted with amoeba isolates and photographed after 30 d of interaction. A) Control colony of P. brasiliensis Pb18 displaying intense filamentation. B) Colony of P. brasiliensis Pb18 co-incubated with A. spelaea. C) Colony of P. brasiliensis Pb18 co-incubated with V. vermiformis. D) Colony of P. brasiliensis Pb18 co-incubated with Acanthamoeba spp. (TIF) [file pntd.0007742.s002.tif]

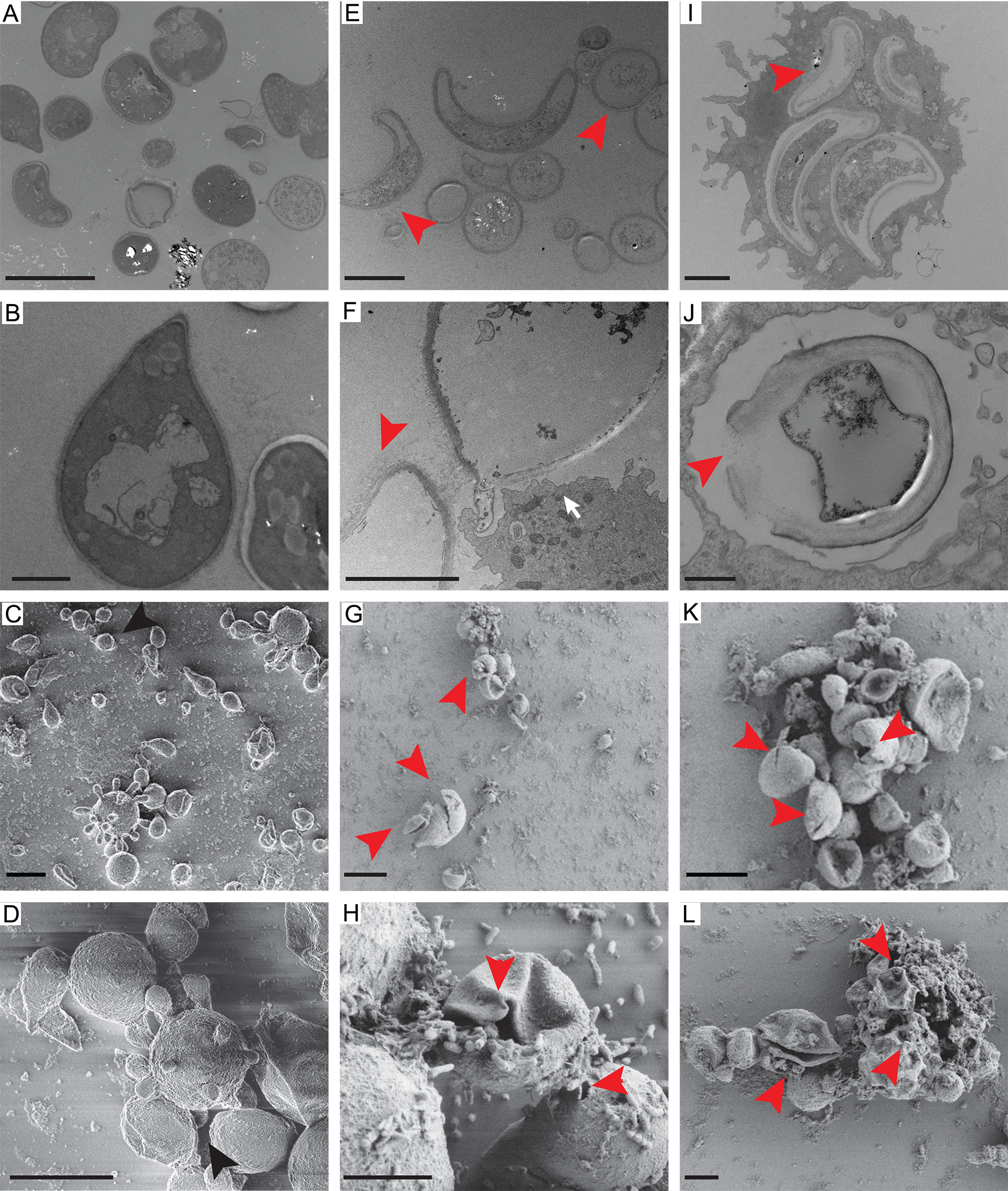

Supplement: S3 Fig — The isolates were co-incubated with Pb18 at an MOI of two at 25°C for 4 or 24 hours in PAS and fixed for TEM or SEM. A-B) TEM of P. brasiliensis cells growing alone. Scale bars = 10 μm. E, F) TEM showing the morphology of P. brasiliensis cells after the interaction with Acanthamoeba spp. Scale bars = 5 μm. I and J) TEM showing the morphology of P. brasiliensis cells after the interaction with A. spelaea, or V. vermiformis, respectively. Scale bars = 5 μm and 500 nm. C-D) SEM of P. brasiliensis cells growing alone. Scale bars = 5 μm and 10 μm, respectively. G and H) SEM showing the morphology of P. brasiliensis cells after the interaction with V. vermiformis or A. spelaea. Scale bars = 10 μm and 5 μm. K, L) SEM showing the morphology of P. brasiliensis cells after the interaction with V. vermiformis or Acanthamoeba spp. respectively. Scale bars = 10 μm and 5 μm, respectively. Red arrowheads indicate fungal cells, or their remains. (TIF) [file pntd.0007742.s003.tif]

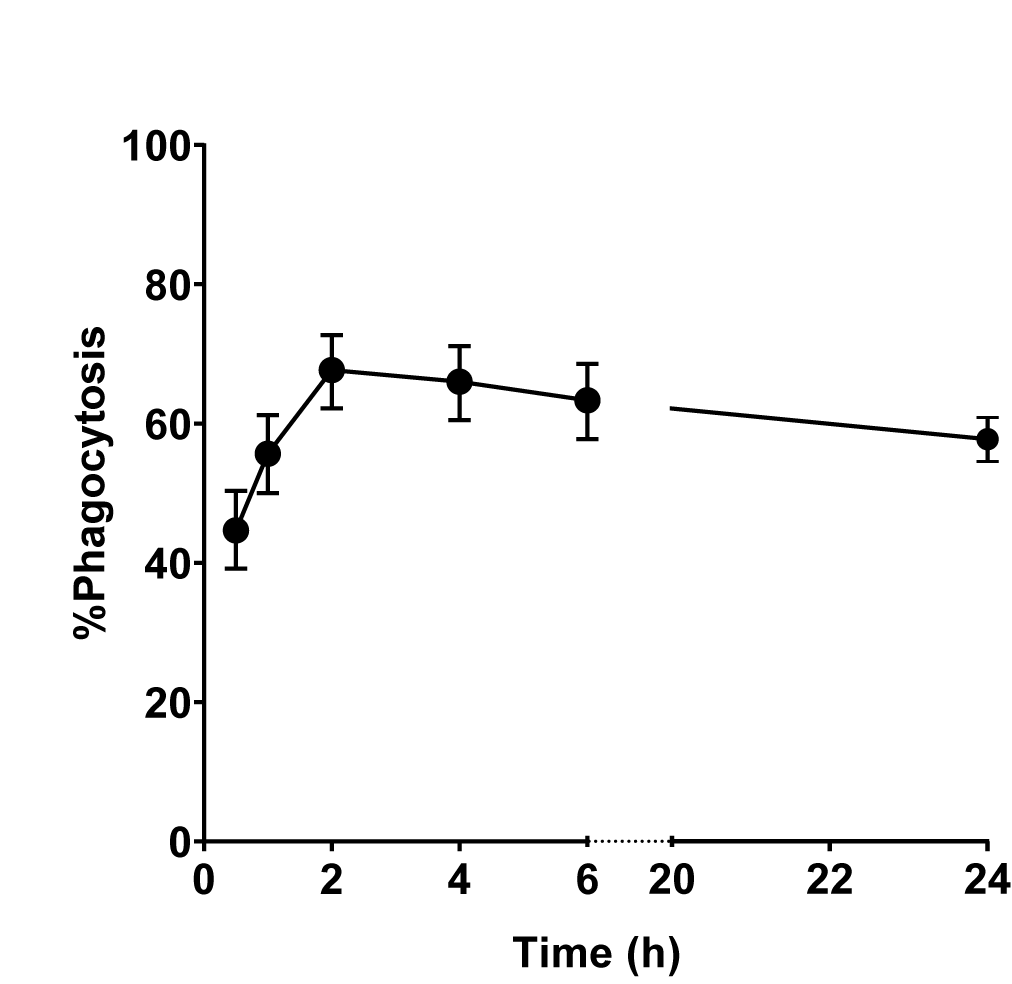

Supplement: S4 Fig — Amoebae and P. brasiliensis yeast cells (CMFDA labeled) were co-incubated (MOI of two). At each time point, the percentage of phagocytosis was evaluated by fluorescence microscopy. A minimum of 300 amoebae per sample was analyzed to calculate the percentage of phagocytosis. The plot represents the results from three independent experiments each performed in triplicate. The error bars represent the 95% confidence interval. (TIF) [file pntd.0007742.s004.tif]

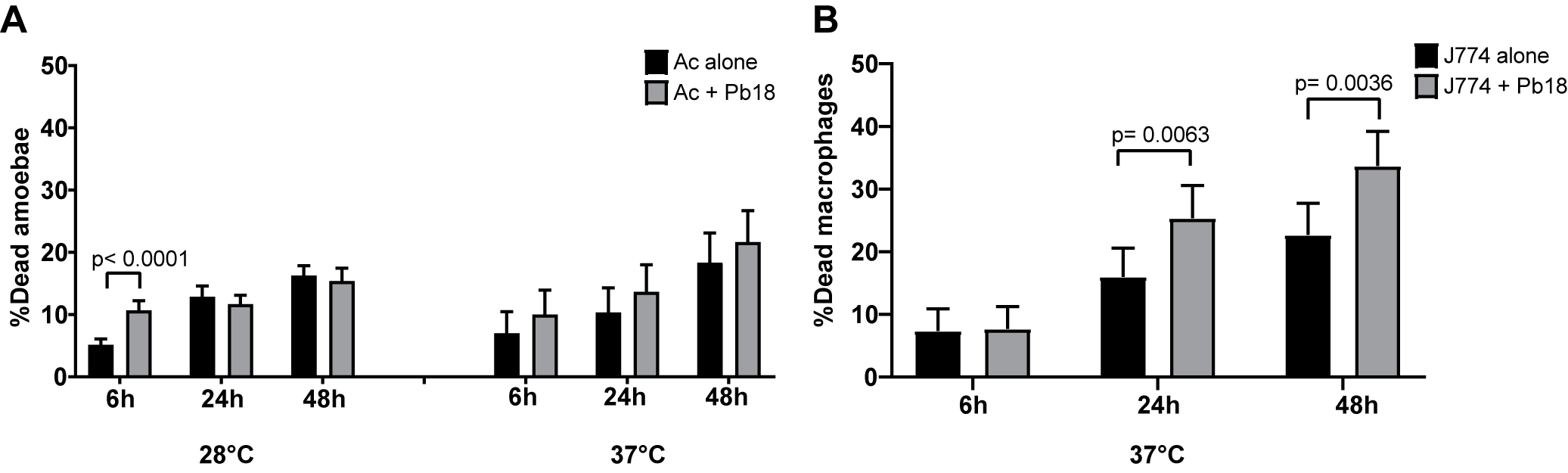

Supplement: S5 Fig — A) Amoeba cells were incubated alone or in the presence of P. brasiliensis at 28°C or 37°C for six, 24 and 48 hours (MOI of two). (B) J774 macrophages were incubated alone or in the presence of P. brasiliensis at 37°C in a CO2 incubator for six, 24 and 48 hours (MOI of two). Viability was assessed at each time point by counting at least 300 phagocytes cells per replicate after staining with trypan blue. The error bars indicate the 95% confidence interval. (TIF) [file pntd.0007742.s005.tif]

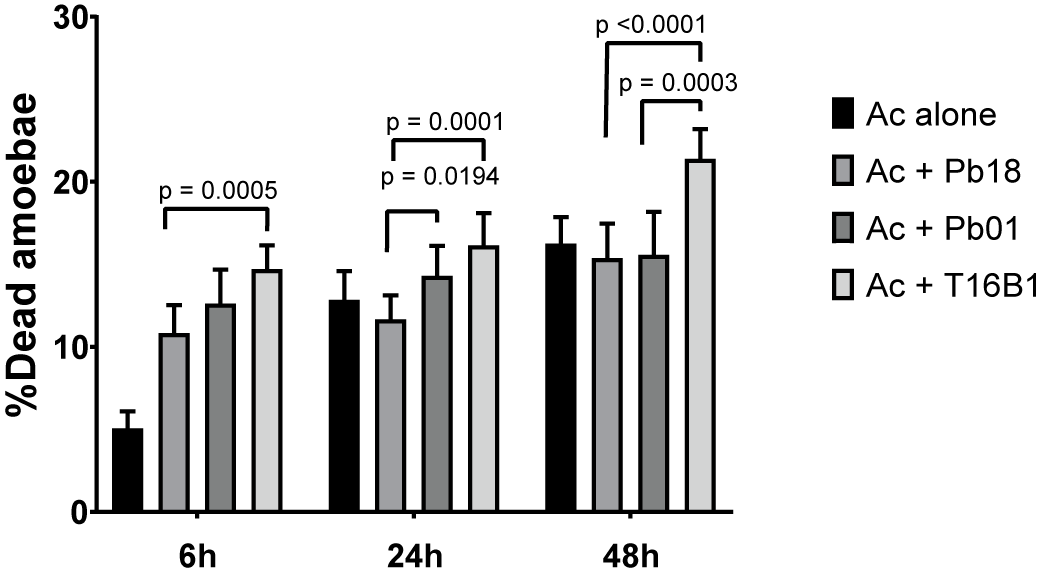

Supplement: S6 Fig — A) Amoebae were incubated alone or in the presence of P. brasiliensis Pb18, P. lutzii Pb01 or P. brasiliensis T16B1 yeast cells at 28°C for six, 24 and 48 hours (MOI of two). Viability was assessed at each time point by counting at least 300 phagocytes cells per replicate after staining with trypan blue. The error bars indicate the 95% confidence interval. (TIF) [file pntd.0007742.s006.tif]
