# Supplementary material for: A hidden battle in the dirt: Soil amoebae interactions with Paracoccidioides spp
Source: PLoS Negl Trop Dis. 2019 Oct 7;13(10):e0007742. doi: 10.1371/journal.pntd.0007742 (PMC6797224; doi:10.1371/journal.pntd.0007742)
Supplement: S1 Table — (DOCX) [file pntd.0007742.s007.docx]

| Gene | Forward Primer | Reverse Primer |
| --- | --- | --- |
| Malate sintase (MS1) (PADG_04702) | TCAACTATCTCATGGAAGATGC | TCAACTATCTCATGGAAGATGC |
| 3-Hidroxi-acil-CoA desidrogenase (HADH) (PADG_01228) | GAGTTCGCCAACAAACTTCTCG | TGATCATGGAGCGGACTTGG |
| Alpha-glucan synthase (AGS1) (PADG_03169) | TCTGTGGCAACCTTGGGAGAC | TCCAGATTACTTGATGCTCAGTG |
| Heat Shock Protein 60 (HSP60) (PADG_08369) | GATTACCAAGGACGGCGTTAC | TCTTGGAGGCAACGTCCTG |
| Heat Shock Protein 70 (HSP70) (PADG_00778) | TTCCTGGCTTGAAACACAGC | AACTCGCGGATTTTCGCTTC |
| Heat Shock Protein 90 (HSP90) (PADG_07715) | ATAAGACGCTGTCCAATGACTG | TTGGGCACGAAGAGGATGG |
| Superoxide dismutase 1 (SOD1) (PADG_07418) | AAGGCCGTCGCTGTTCTC | CTGTATGTGATGACGGTTGCG |
| 60S Ribosomal protein L34 (L34)  (PADG_04402) | CTCCCGCGAATCCACAAC | ATGTGTTGGTGGGAGAGGAG |
