# Supplementary material for: A hidden battle in the dirt: Soil amoebae interactions with Paracoccidioides spp
Source: PLoS Negl Trop Dis. 2019 Oct 7;13(10):e0007742. doi: 10.1371/journal.pntd.0007742 (PMC6797224; doi:10.1371/journal.pntd.0007742)
Supplement: Attachment — Submitted filename: Response letter PNTD-D-19-00966.docx [file pntd.0007742.s008.docx]

Baltimore, August 20, 2019.

Dear Dr. Reynolds

We are very pleased with the positive reviews and suggestions to our paper "A hidden battle in the dirt: soil amoebae interactions with *Paracoccidioides* spp" (PNTD-D-19-00966). We have addressed all their questions and suggestions, as presented in a point by point response below. To facilitate reading, our responses are in blue.

Reviewer's Responses to Questions

**Key Review Criteria Required for Acceptance?**


As you describe the new analyses required for acceptance, please consider the following:


**Methods**


-Are the objectives of the study clearly articulated with a clear testable hypothesis stated?

-Is the study design appropriate to address the stated objectives?

-Is the population clearly described and appropriate for the hypothesis being tested?

-Is the sample size sufficient to ensure adequate power to address the hypothesis being tested?

-Were correct statistical analysis used to support conclusions?

-Are there concerns about ethical or regulatory requirements being met?


Reviewer #1: The methods are clear, concise and well designed to address the study objective. The sample size was enough to ensure the results were statistically valid. Ethical requirements were met.

Response: We appreciate the reviewer’s comment.

It would be good if the authors give references for all their methods i.e. electron microscopy (EM) work, etc.
For EM, it would have been good to include a control of cryptococcal cells alone to exclude the harsh preparatory steps of EM as the reason for collapsed cells.

Response: We included references for the methods we used (page 8, line 208 and page 9, line 221). We transferred methodological details that were previously described in the Supplementary Material and Methods to the main text to clarify the experimental approach. Regarding the suggested control, it was actually part of the originally submitted manuscript (supplementary figure 3). In this figure, we show that *P. brasiliensis* cells (not cryptococcal cells) grown alone do show some signs of damage that are possibly caused by the preparation protocol, but the pattern is significantly different from that observed in fungal cells that were co-incubated with amoebae. We have modified the text (page 8, lines 201-202 and pages 13-14 lines 336-339) to make it clearer that this control had been done.

Reviewer #2: Yes.

Response: We appreciate the reviewer’s comment.

Reviewer #3: The authors should not include results in the Introduction section "These studies showed that amoebae efficiently ingest and kill Paracoccidioides spp. yeast cells, and that this interaction selects for fungi with increased virulence. Our results support the hypothesis that interaction with neighbor soil predators selects for traits that allow survival of Paracoccidioides spp. in mammalian hosts and add to the existing evidence for the amoeboid predator-animal virulence hypothesis [13].".

Response: We agree with the reviewer’s comment and we suppressed this information from the manuscript introduction.

The objectives of the study are clearly articulated

The methods were described with sufficient detail and there is information to replicate.

Response: We appreciate the reviewer’s comment.

--------------------

**Results**


-Does the analysis presented match the analysis plan?

-Are the results clearly and completely presented?

-Are the figures (Tables, Images) of sufficient quality for clarity?


Reviewer #1: The analysis match the analysis plan. However, lack of a control for EM work questions the findings.

Response: The EM controls had actually been presented in Figure S3. We change the text to make this information clearer to the reader.

To harvest the cryptococcal cells, amoeba had to be lysed. Could the latter see amoeba also contributing to the observed levels of glucans?

Response: *Paracoccidioides* spp (not cryptococcal cells) were grown in BHI agar plates for about a week after the interaction with amoebae. In addition to that, fungal cells were washed in PBS for 3 times before evaluation of alpha-glucan levels in the fungal cell wall. We thus believe that interference of amoeba glucans in this experiment is unlikely. We clarified this information in the material and methods section (page 11, lines 271-273).

How did the authors normalize the mRNA levels of genes of interest?

Response: Fungal mRNA levels were normalized against the L34 transcript, which was used as an endogenous control. This transcript is highly expressed in *Paracoccidioides* yeast cells and has been successfully used by several groups before as an endogenous control. This information was in the supplementary material and methods in the original manuscript and was moved to the Material and Methods section (page 11, lines 264-265) to improve clarity. We also included a reference for the use of L34 as an endogenous control for gene expression studies of *Paracoccidioides* spp.

Reviewer #2: Yes.

Response: We appreciate the reviewer’s comment.

Reviewer #3: The results are clearly and completely presented, and the figures are sufficient quality.

Response: We appreciate the reviewer’s comment.

--------------------

**Conclusions**


-Are the conclusions supported by the data presented?

-Are the limitations of analysis clearly described?

-Do the authors discuss how these data can be helpful to advance our understanding of the topic under study?

-Is public health relevance addressed?


Reviewer #1: The public health relevance is adequately addressed. The conclusions that reached confirm what is already known in literature.

Response: We appreciate the reviewer’s comment.

Reviewer #2: Could be modified.

Response: We changed the conclusion section to better address the limitations of our approach (page 18-19, lines 473-476, page 19-20, lines 495-504).

Reviewer #3: The limitations of study were not described. I suggest Include and discuss the limitations of your study.

Response: In our original manuscript we have addressed a number of limitations from our study in the results section along with the experimental approaches we used to overcome some of them. These limitations include: a) the decision to focus on ameboid predators despite a number of other potential predators that were co-isolated with them (page 12, lines 296-298); b) the impossibility of obtaining axenic cultures of soil amoebae (page 13, lines 314-318); c) our inability to perform more quantitative analysis of fungal survival after the interaction with the isolated soil amoeba due to presence of contaminating bacteria (page 13, lines 315-319); d) the need to use of a laboratory adapted strain of *A. castellanii* for part of the experiments (page 14, lines 347-349). We included a paragraph about those limitations in the discussion section (page 18-19, lines 473-476, page 19-20, lines 495-504).

**Editorial and Data Presentation Modifications?**


Use this section for editorial suggestions as well as relatively minor modifications of existing data that would enhance clarity. If the only modifications needed are minor and/or editorial, you may wish to recommend “Minor Revision” or “Accept”. 

Reviewer #1: Minor revision

Reviewer #2: Lines 186-187: the original strain is part of the comparison, so not a control. Could be “with strains Pb18-Ac or the non-passaged form Pb18.”

Response: We agree and changed the text accordingly (page 9, line 233).

Line 203: space “glucan, and”.

Response: We agree and changed the text accordingly (page 11, line 274).

Line 206: “analyses” plural.

Response: We agree and changed the text accordingly (page 11, line 278).

Line 234: “hits at GenBank”.

Response: We agree and changed the text accordingly (page 12, line 306).

Line 274: “were” [bacteria plural].

Response: We agree and changed the text accordingly (page 14, line 347).

Line 354: “through mechanisms other than mRNA accumulation”.

Response: We agree and changed the text accordingly (page 16, line 422).

Line 386: “1970s”.

Response: We agree and changed the text accordingly (page 18, line 454).

References: check the formatting is correct, as the journal will be unlikely to do so, e.g. italics on species or gene names.

Response: We have thoroughly checked the references once more.

Lines 728-729: change to be “Figure 7- Changes in P. brasiliensis Pb18 gene expression after cycles of interaction with amoeba. Transcript accumulation…” the first sentence in bold.

Response: We agree and changed the text accordingly (page 23, line 615).

Reviewer #3: “Minor Revision”

--------------------

**Summary and General Comments**


Use this section to provide overall comments, discuss strengths/weaknesses of the study, novelty, significance, general execution and scholarship. You may also include additional comments for the author, including concerns about dual publication, research ethics, or publication ethics. If requesting major revision, please articulate the new experiments that are needed.

Reviewer #1: The presented speak to the idea that amoebal predatory pressure may select Paracoccidioides to become pathogenic. This (selection) has also been observed elsewhere for other fungal species. Therefore, there is no novelty in the work. Nonetheless, the work still highlights the medical importance of the fungus and acquisition of its virulence. 

Response: We understand the reviewer’s point, but none of the previous studies included *Paracoccidioides* spp. Most importantly, previous studies were made exclusively with laboratory-adapted amoeba that had been grown axenically for decades. These previous studies assumed that *A. castellanii* or *D. discoideum* would be found in the same environment and that adaptation to laboratory cultivation did not change the predatory ability of amoebae against fungi. Our study with amoeba species that probably share the same microenvironment as *Paracoccidioides* is thus a significant advance for the field. One example of an insight that came with our approach is that smaller amoebae seem to feed on larger fungal yeast cells by puncturing their cell walls instead of phagocytosing them. Given that this is the antifungal mechanism used by immune cells such as T CD8 and NK cells, our work suggests that amoebae might also have selected fungi to resist other mammalian immune cells besides phagocytes.

The authors should consider sufficiently addressing the issues that are raised.

Response: We have thoroughly revised the manuscript to address the issues raised. We believe this undoubtedly improved it.

Reviewer #2: The manuscript reports on the interaction of Paracoccidioides strains with three amoeba species isolated from armadillo burrows, which are associated with Paracoccidioides. Passage of fungal isolates through amoeba lead to ‘evolved’ strains with increased virulence in both mice and an insect model, Galleria mellonella, leading to support of the hypothesis that amoeba (or other predators) are important in maintaining the virulence of Paracoccidioides when it encounters humans.

This is a substantial body of work. One reflection of this is that mouse studies went for 12 months. The electron microscopy adds the visual appeal of the work, and the level of replication to infer statistical significance is noteworthy. The work will appeal to the medical mycology community, especially those working on pathogens that have environmental reservoirs. Probably the main point of concern is how the results are interpreted overall.

Response: This manuscript is indeed the product of eight years of work. We thank the reviewer for the comments.

1) ‘in controlled laboratory conditions using mostly pure and axenic cultures of
laboratory-adapted predators; this very informative system is nonetheless an extreme
simplification of the complex ecosystem soil saprophytes find in nature’ is a very valid point, and it is relevant that here new amoeba strains were isolated and characterized for their interaction with Paracoccidioides strains. However, counter to this the authors then rely on the standard A. castellanii strain ATCC 30234 [which was identified as a natural predator of another pathogenic fungus, Cryptococcus neoformans, so not as artificial as other amoeba strains] for subsequent characterization of the impact of amoeba on Paracoccidioides by passaging.

Response: We made multiple attempts over a period of 4 years to obtain pure cultures of the soil amoebae for the passage experiments. We tried using differential centrifugation, several different antibiotics alone or in combination, non-nutrient media, treatment with hydrochloric acid and even a passage of amoebae in macrophage cultures, all to no avail. We did carry on *Paracoccidioides* passage experiments with these amoebae. However, all experiments were ruined by bacteria present on the amoeba cultures, which overgrew the slow-growing *Paracoccidioides* yeast cells. We realize the limitations of the approach we took and we revised how this is discussed in the manuscript (page 18-19, lines 473-476, page 19-20, lines 495-504). However, we still believe our results support the conclusion that predation by amoebae plays a role in the evolution of virulence in the genus *Paracoccidioides*.

2) ‘Sequential co-cultivation of Paracoccidioides with A. castellanii selected for
phenotypical traits related to the survival of the fungus within a natural predator as well as in
murine macrophages and in vivo (Galleria mellonella and mice). This increase in virulence…’ However; Pb18 is already an attenuated strain, so while certainly the passaging has changed the virulence properties, it is unclear that this is not just back to the baseline level.

Response: We agree with the reviewer. The non-passaged strain we used indeed showed virulence attenuation considering the results from the infection of different animal models. Given the many years these cultures have been propagated in our laboratory, it is impossible to compare the virulence of passaged strains with the baseline virulence of the original isolates. We have revised the entire manuscript to use more precise language in our interpretation of the changes in virulence after passage as a comparison to the non-passaged strain we used.

3) A point that needs addressing more explicitly, counter to statements like “the higher susceptibility of Paracoccidioides to amoeba”, is that some survive predation to become more fit in causing disease. The data (such as figure 2) show that a considerable proportion of the fungal isolates survive.

Response: In this instance, we actually compared our results with those of experiments made in the early 2000’s with other fungal pathogens such as *C. neoformans*, *Histoplasma capsulatum*, *Sporothrix schenckii* and *Blastomyces dermatitidis*. These fungal pathogens were highly efficient in killing *A. castellanii* and in surviving the interaction, in contrast to the results we obtained. The figure pointed by the reviewer (Fig. 2) actually depicts amoeba survival. In figure 4, we show that we still have many fungal cells alive after 6h of interaction with *A. castellanii* (panel E), but almost no fungal colonies are obtained after 24h of interaction. That was the reason we have chosen cycles of 6 h of interaction for the passage assay. We have revised the manuscript text to be clearer and more precise in our interpretation of the data.

Reviewer #3: The manuscript "A hidden battle in the dirt: soil amoebae interactions with Paracoccidioides spp." is well written in an engaging and the level is appropriate to readership of PLoSNTD. I have no hesitation in recommending that it be accepted for publication after a few minor details have been attended to, without the need for further experimental work.

Response: We thank the reviewer for the praise.
--------------------

PLOS authors have the option to publish the peer review history of their article ([what does this mean?](https://journals.plos.org/plosntds/s/editorial-and-peer-review-process#loc-peer-review-history)). If published, this will include your full peer review and any attached files.


If you choose “no”, your identity will remain anonymous but your review may still be made public.


**Do you want your identity to be public for this peer review?** For information about this choice, including consent withdrawal, please see our [Privacy Policy](https://www.plos.org/privacy-policy).

Reviewer #1: No

Reviewer #2: No

Reviewer #3: No

__________________________________________________
In compliance with data protection regulations, you may request that we remove your personal registration details at any time. (Use the following URL: https://www.editorialmanager.com/pntd/login.asp?a=r). Please contact the publication office if you have any questions.
